# Modern India and Dietary Calcium Deficiency—Half a Century Nutrition Data—Retrospect–Introspect and the Road Ahead

**DOI:** 10.3389/fendo.2021.583654

**Published:** 2021-04-06

**Authors:** Chittari Venkata Harinarayan, Harinarayan Akhila, Edara Shanthisree

**Affiliations:** ^1^ Institute of Endocrinology, Diabetes, Thyroid and Osteoporosis Disorders, Sakra World Hospitals, Bangalore, India; ^2^ Department of Medicine & Endocrinology, Saveetha Medical College, Saveetha Institute of Medical and Technical Sciences University, Chennai, India; ^3^ IT Industry—Digital Transformation, Information Services Group (ISG), Bangalore, India

**Keywords:** modern India, recommended dietary allowances, recommended dietary intake, vitamin D, dietary calcium deficiency, fortification, Ragi (Eleusine coracana), production supply consumption chain

## Abstract

Calcium and vitamin D are inseparable nutrients required for bone health. In the past half a century, the dietary calcium intake of rural, tribal, and urban India has declined. Though India is the largest producer of milk and cereals, the major source of calcium in India is through non-dairy products. The highest intake of cereals and lowest intake of milk & milk products was observed in rural and tribal subjects whereas, the intake of cereals, milk & milk products were similar in both urban and metropolitan subjects. One of the reasons for lower calcium intake was the proportion of calcium derived from dairy sources. Over the past half a century, the average 30-day consumption of cereals in the rural and urban population has declined by 30%. The Per Capita Cereal Consumption (PCCC)has declined despite sustained raise in Monthly Per capita Consumption Expenditure (MPCE) in both rural and urban households. The cereal consumption was the highest in the lowest income group, despite spending smaller portion of their income, as cereals were supplied through public distribution system (PDS). About 85% of the Indian population are vitamin D deficient despite abundant sunlight. Dietary calcium deficiency can cause secondary vitamin D deficiency. Though India as a nation is the largest producer of milk, there is profound shortage of calcium intake in the diet with all negative consequences on bone health. There is a decline in dietary calcium in the background of upward revision of RDI/RDA. There is a gap in the production-consumption-supply chain with respect to dietary calcium. To achieve a strong bone health across India, it is imperative to have population based strategies addressing different segments including supplementing dietary/supplemental calcium in ICDS, mid-day-meals scheme, public distribution system, educational strategies. Other measures like mass food fortification, biofortification, bioaddition, leveraging digital technologies, investments from corporate sector are some measures which can address this problem. India is a vast country with diverse social, cultural and dietary habits. No single measure can address this problem and requires a multi-pronged strategic approach to tackle the dietary calcium deficiency to achieve strong bone health while solving the problem of nutritional deficiency.

## Introduction

Billions of years ago, life originated in the primordial seas near volcanic craters where the ionic calcium concentration of the ocean was stable, at about 10 mM. During the course of evolution, calcium became more intracellular for complex cellular functioning (1–3). The evolution of parathyroid hormone (PTH) and vitamin D endocrine system regulated calcium homeostasis and phosphatonin-FGF23 which is the major regulator of serum phosphate homeostasis (4, 5).

Vitamin D is found in phytoplankton and zooplankton. Phytoplankton is a major part of food chain of many fish (6–12). From amphibian onwards, vitamin D endocrine system is an important regulator of calcium (for the calcified skeleton) while moving from calcium-rich ocean to calcium-poor terrestrial environment. Evolutionally, this coincides with creation of bone cells-osteocytes which regulate mineral homeostasis, mechanical sensing and production of hormones. The fully developed vitamin D endocrine system comprises of specific nuclear receptor (NR), enzyme C-450 family which metabolizes vitamin D and specific high affinity, high capacity vitamin D binding protein for extracellular transport system (11, 12).

The lifestyle of homo-sapiens transited from a hunter-gatherer, to animal and plant domesticator 12-8 k.y.a (thousands of years ago), while transiting from Paleolithic to Neolithic period (13). For the Neolithic humans, cow milk provided twice as much calcium than their Paleolithic predecessors (14). During the agrarian revolution, cereals and legumes became major source of calcium and energy, however, they were rich in anti-nutrients, phytates, oxalates which diminish calcium absorption (15, 16). The agrarian revolution occurring at various locations was associated with crowding of people in cities and increase in dietary calcium. In western countries, milk has been a major source of calcium (35%–70% of dietary calcium) (17).

## Calcium-Vitamin D Endocrine System

**Figure 1:**
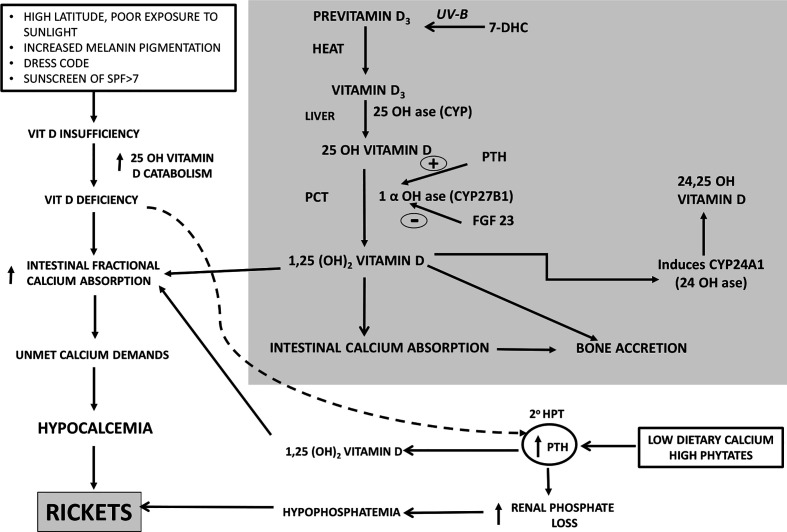
Calcium-Vitamin D-PTH endocrine axis in health during calcium and/or vitamin D deprivation. PTH, Parathyroid Hormone, 2^0^ HPT, secondary hyperparathyroidism, SPF, Skin Protection Factor.

Vitamin D_3_ synthesized in the skin undergoes successive sequential hydroxylations in the liver and kidney to be converted to its active form1,25 dihydroxyvitamin D_3_(1,25OHD_3_) which helps in calcium absorption from the gut and mineralization of the skeleton. The secondary hyperparathyroidism(SHPT) which is an accompaniment of low dietary calcium leashes the bones to maintain normal serum calcium. This SHPT leads to increased catabolism of 25 Hydroxyvitamin D (25OHD) leading to secondary vitamin D deficiency (**Figure 1**) **(** 18, 19). Thus, vitamin D is a key factor adjusting the demand/supply of calcium at adolescent growth spurt or at weaning, whenever dietary calcium intake may fall or bone accretion rates may maximize. Calcium absorption during pregnancy is possibly dependant on estrogens and appears to be independent of vitamin D (20).

Nutritional rickets is a major global public health problem which has an impact on health of infants, children, adults and ramifications persist into adulthood. Nutritional rickets (NR) can be due to calcium deficiency and/or secondary to vitamin deficiency (**Figure 1**). The clinical features associated with NR are: a) *non osseous features*- poor linear growth and failure to thrive, muscle weakness, delayed motor development, tetany, hypocalcemic seizures, hypocalcemic dilated cardiomyopathy and raised intracranial pressure; b) *osseous symptoms and signs*- bone pains, craniotabes, frontal bossing, delayed closure of fontanelle and eruption of tooth, rachitic rosary, swelling of wrist and ankles, leg deformity; c) cupping, fraying, splaying of metaphysis, widening of growth plates, low bone mineral density(BMD), pseudo fractures, pelvic deformities, pelvic outlet narrowing (19, 20).

In many developing countries, across tropical and subtropical regions, NR remains a major health problem amongst infants, young children, adolescents. Lack of sunlight exposure, changing lifestyles(use of SPF>7), social customs limiting skin exposure to sunlight, atmospheric pollution remain the leading causes of vitamin D deficiency. There are reports of rickets developing in children with low calcium intake. The dietary intake of calcium of these children in developing countries is ~1/3–1/2 of Recommended Dietary Intake(RDI) (20). When the calcium intake is low, body adapts by increasing intestinal fractional absorption and reducing renal calcium excretion. When vitamin D insufficiency is associated with low dietary calcium intake, they act synergistically to exacerbate development of rickets (**Figure 1**). Lack of dietary calcium for prolonged duration in adults leads to low BMD. This is more pronounced in population where the inadequacy of calcium persists from childhood, adolescent and adults leading to low peak bone mass.

## Methodology

In this presentation, we review the nutritional status of India in the past half century, changes in milk and cereal production and consumption while documenting trends in consumption expenditure. The article also emphasizes the interaction between vitamin D and calcium deficiencies and point to inadequacies in the recent Recommended Dietary Allowances (RDA) of Indian council of Medical research and Nation institute of Nutrition (ICMR-NIN). Second part of the article emphasizes existing strategies by the government of India, their shortfall and the remedial measures. Role of e-health and m-health in combating these problems are discussed.

## Historical Perspective of Nutritional Status in India—Post Independence

In the second half of 18^th^ century, in the West, beginning of industrial revolution was associated with rickets due to industrial air pollution. With transition to digital age, there is further diminution of UV exposure in humans. In the late 19^th^ and early 20^th^ century, more than 80% of children living in the West had growth deformities and features of rickets (21). During this period, the West went through industrial revolution, while India was suffering due to poverty and famine. Post-independence(1947), millions of households did not have even two square meals a day. The immediate goal for the country was to achieve availability of cereals. With this background, we analyze the nutritional status of India in post-independence era. This analysis emphasizes the dietary calcium deficiency of various age groups in different regions of India and their dietary habits.

## Nutrition Trends India 1957–2017

### Rural Survey: National Nutrition Monitoring Bureau

**Figure 2:**
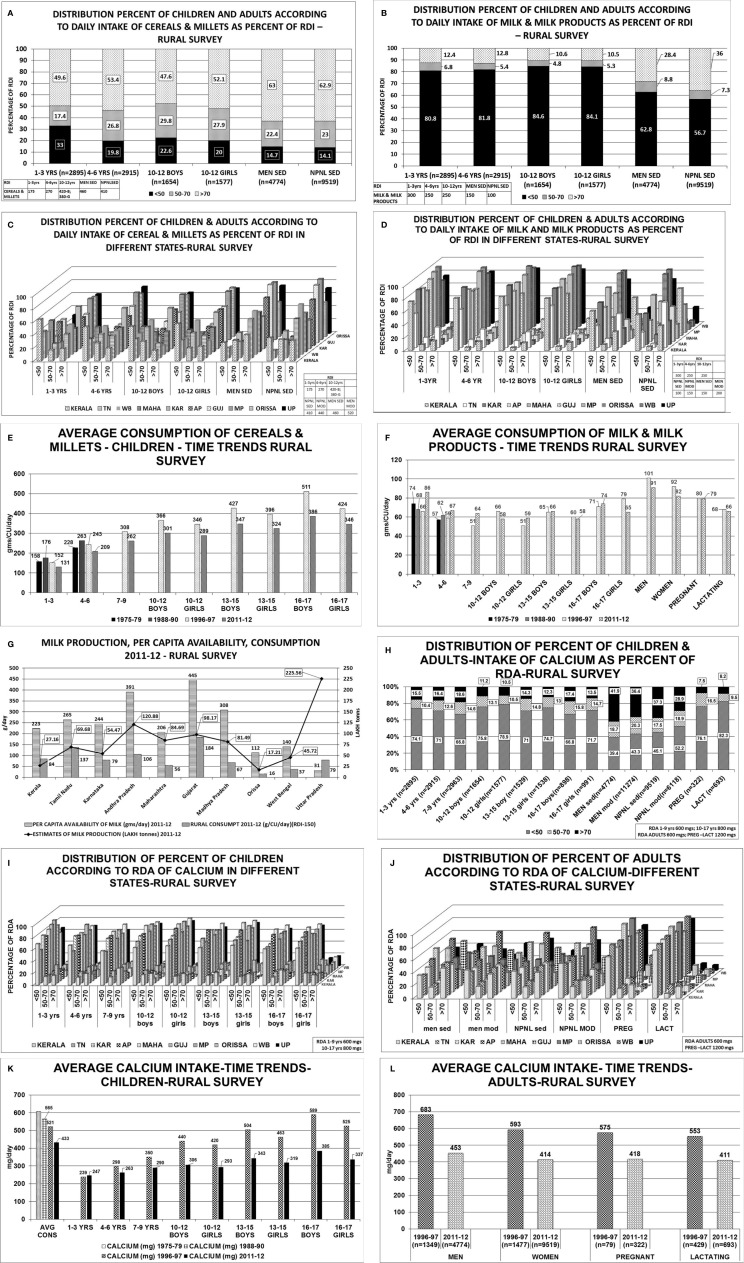
Rural survey (**Table 1**) (22): **(A)** Distribution percent of children and adults according to daily intake of cereals & millets as percent of RDI. B Distribution percent of children and adults according to daily intake of milk & milk products as percent of RDI. **(C)** Distribution percent of children and adults according to daily intake of cereals & millets as percent of RDI in different states. *children 1–3 years* consumed >70% of RDI. The highest consumption of >70% of RDI was seen in Karnataka, MP, Orissa, 61%–65% in UP, 36%–45% in TN, AP, Gujarat, WB and only 19% in Kerala. Similar trend was seen in *children with 4–6 years, 10–12 years boys & girls*. More than 2/3 of *men* from Karnataka, AP, Gujarat, Orissa, UP consumed >70% of RDI of cereals & millets, 50-60% in TN, Maharashtra, WB and only 40% of subjects from Kerala. Similarly, >60% of *women* from TN, Karnataka, AP, Gujarat, MP, Orissa, UP consumed >70% of the RDI and the lowest was 31% from Kerala. **(D)** Distribution percent of children and adults according to daily intake of milk & milk products as percent of RDI in different states. *1–3 years children*- 75-95% of children from Karnataka, AP, Gujarat, MP and Orissa consumed <50% of the RDI, except TN-with 53%. Less than 10% of children from the above states consumed >70% of RDI(TN-31.8%). Only 0.4% of children from Orissa consumed >70% of milk & milk products. Similar trend was seen in *children 4–6 years, 10–12 years boys & girls.* 65%–90% of *men* consumed <50% of RDI of milk & milk products. More than 70% of RDI consumption was seen in TN(53%), Gujarat(59%), Kerala(29%). **(E)** Average consumption of cereals & millets- Time trends showing a decline in consumption of cereals & millets by 20% in the past 4 decades. **(F)** Average consumption of milk & milk products- Time trends showing a decline in consumption of milk & milk products in the past 4 decades. **(G)** Graph depicting milk production and per capita availability for year 2011–2012. The consumption data of the rural survey for year 2011–2012 is superposed. Graph clearly depicts the low consumption of milk despite adequate availability. **(H)** Distribution of percent of children & adults–intake of calcium as percent of RDA. **(I)** Distribution of percent of children according to RDA of calcium in different states. Between 70%–85% of children, adolescent boys & girls of all the states have low calcium intake (<50% of RDA). Less than 15% of children (1–3 years) from Kerala, Karnataka, AP, Maharashtra, Gujarat, MP, Orissa, and 25%–30% of children from TN, WB and UP had intake of >70% of RDA. Similar pattern was seen in *4–6 years, 10–12 years boys & girls age group*, only 10%–12% of subjects from all the states of *age group 13–15 years boys & girls* had an RDA of calcium >70%. (J) Distribution of percent of adults according to RDA of calcium in different states. *Sedentary men*-51 and 65% of subjects from TN, Gujarat and 25%–35% of subjects from AP, Maharashtra and Orissa had a calcium intake of >70% of RDA. *Moderately active men*->70% of RDA of calcium was met with by 35%–42% of the subjects from all states except Gujarat(50.8%), Maharashtra and WB (21%). Amongst the *non-pregnant women* 35%–45% of subjects from all states, except Gujarat (55%) and WB (27%), met with >70% of RDA of calcium. Uniformly, only 25%–35% of subjects of moderately active *non-pregnant women* met with adequate RDA of >70% with exception of Maharashtra (19%) and WB (16%). Less than 10% of pregnant women of all states except Kerala (21%) met with RDA limit of >70%. 65%–75% of them had RDA of <50% except Maharashtra (97%) and MP (96%). Similar scenario seen in lactating women. **(K)** Average calcium intake children time trends showing a decline in calcium intake over the past 4 decades. **(L)** Average calcium intake adults time trends showing a decline in calcium intake over the past 4 decades. RDI-Recommended Daily Dietary Intake; RDA-Recommended Daily Dietary Allowance; HH- Households; B-Boys; G-girls; WOMEN NPNL SED-women: non-pregnant, non-lactating and sedentary; WOMEN NPNL MOD-women: non-pregnant non-lactating and moderate; WOMEN PREG SED-women: pregnant and sedentary; WOMEN PREG LACT-women: pregnant and lactating; MEN SED-men: sedentary; MEN MOD-men: moderate; TN-Tamil Nadu; WB-West Bengal; MAHA-Maharashtra; KAR-Karnataka; AP-Andhra Pradesh; GUJ-Gujarat; MP-Madhya Pradesh; UP-Uttar Pradesh.

The consumption of cereals, millets, milk & milk products across all age groups was below the RDI among children, adolescents, men and women-non pregnant, pregnant, lactating and elderly (**Table 1**; **Figure 2**) (22, 23) The state wise distribution is detailed in **Figures 2A–L**. The milk production per capita availability and consumption for the corresponding period of the survey (2011–2012) is shown in **Figure 2G**.

**Table 1:** Table depicting RDI of cereals, millets, milk and milk products, and RDA of calcium intake across various age groups in rural survey (22, 23) and tribal survey (24).

GROUP	CEREALS & MILLETS				MILK & MILK PRODUCTS				CALCIUM INTAKE			
	AVG INTAKE	RDI	<50% RDI	≥70% RDI	AVG INTAKE	RDI	<50% RDI	≥70% RDI	MEDIAN CAL INTAKE	RDA	<50% RDA	≥70% RDA
**RURAL STUDY (** 22, 23)												
1–3 YRS	131	175	33	49.6	86	300	80.8	12.4	166	600	74.1	15.5
4–6 YRS	209	270	19.8	53.4	67	250	81.8	12.8	198	600	71	16.4
7–9 YRS	262	–	–	–	64				226	600	66.8	18.6
10–12 GIRLS	289	380	20	52.1	59	250	84.1	10.5	230	800	78.9	10.5
10–12 BOYS	301	420	22.6	47.6	58	250	84.6	10.6	248	800	75.8	11.2
13–15 BOYS	347	–	–	–	66	–	–	–	266	800	71	14.3
13–15 GIRLS	324	–	–	–	58	–	–	–	249	800	74.7	12.3
16–17 BOYS	386	–	–	–	74	–	–	–	299	800	66.8	17.4
16–17 GIRLS	346	–	–	–	65	–	–	–	270	800	71.7	13.5
WOMEN NPNL SED	341	410	14.1	62.9	82	100	56.7	36	328	600	45.1	37.3
WOMEN NPNL MOD	391	440	–	–	73	150			292	600	52.2	28.9
WOMEN PREG SED	354	–	–	–	79	–	–	–	334	1200	76.1	7.5
WOMEN PREG LACT	395	–	–	–	66	–	–	–	327	1200	82.3	8.2
MEN SED	380	460	14.7	63	91	150	62.8	28.4	370	600	39.4	41.9
MEN MODER	444	520	–	–	78	200			335	600	43.3	36.4
60–69 YRS MEN*	412	460	<70% of RDI 48.4%	>70% of RDI 51.6%	92	150	<70% of RDI 74.3%	>70% of RDI 25.7%	368	400	<70% RDA 36.8%	>70% RDA 62.2%
70–79 YRS MEN*	374	460			73	150			338	400		
≥80 YRS MEN*	329	460			103	150			287	400		
60–69 YRS WOMEN*	337	420	<70% of RDI 55%	>70% of RDI 45%	76	100	<70% of RDI 66.5%	>70% of RDI 33.5%	290	400	<70% RDA 49%	>70% RDA 51%
70–79 YRS WOMEN*	305	420			77	100			280	400		
≥80 YRS WOMEN*	251	420			76	100			254	400		
**TRIBAL STUDY (** 24)												
1–3 YRS	149	175	20.1	61.9	16.6	300	97.3	1.4	95	400	79.9	10.6
4–6 YRS	231.2	270	12.5	65.6	13.7	250	97.8	0.9	132	400	71.1	16.1
7–9 YRS	289.1				14.5				159	400	63.3	21.4
10–12 BOYS	331.6	420	15.2	59.7	16.1	250	97.1	1.3	171	600	75.7	14.2
10–12 GIRLS	322.6	380	10.6	68.6	16.5	250	96.6	1.6	173	600	77.4	12.1
13–15 BOYS	386.8				20.1				196	600	72.8	16.8
13–15 GIRLS	359				16.1				179	600	74.6	15.3
16–17 BOYS	440.1				22.2				213	500	69.3	19
16–17 GIRLS	383.6				19				203	500	67.2	19.9
WOMEN NPNL SED	377.5	410	8.6	72.3	21	100	84.7	9.5	214	400	46.3	35.3
WOMEN PREG SED	388.2				26.7				204	1000	81	10.1
WOMEN LACT SED	436.4				16.5				230	1000	83.1	10.8
MEN SED	435.9	460	9.2	74.7	24.7	150	90.1	5.9	232	400	40.7	40.6

Rural Survey: Cereals and millets: About 49.6%–63% of them consumed >70% of the RDI according to their age and gender. About 14%–55% consumed <50% of the RDI. About 45%–52% of elderly adults, 60–≥80 years of age, consumed >70% of RDI. Milk & milk products: About 80%–85% of children of all age groups and 57%–63% of adults consumed <50% of the RDI. Only 10%–13% of children, 36% men, and 28% of women consumed >70% of the RDI for their age and gender. About 26% of men and 33.5% of women, between 60–≥80 years of age consumed >70% of RDI. RDA-Calcium: The median intake of calcium was less than RDA for their age and gender. About 67%–79% of children of various age groups, 40%–52% of women and men, 76%–82% of pregnant and lactating women consumed <50% of RDA. Only 10%–17% of children, 29%–42% of men and women;7.5%–8% of pregnant and lactating women consumed >70% of RDA. About 62% men and 51% of women between 60–≥80 years of age had intake of >70% of RDA.

Tribal survey: Cereals: About 60%–75% of them consumed >70% of the RDI for their age and gender. About 9%–20% consumed <50% of the RDI. Milk and milk products: About 85%–98% of children and adults consumed <50% of the RDI. Only 1%–9% of children, men and women consumed >70% of the RDI for their age and gender. RDA-Calcium: The median intake of calcium was less than RDA for their age and gender. About 63%–80% of children of various age groups, 40%–46% of men and women, 81%–83% pregnant and lactating women consumed <50% of RDA. About 10%–21% of children, 35%–40% of women and men, 10% of pregnant and lactating women consumed >70% of RDA. *refers to data from ref (23).

### TRIBAL SURVEY-NNMB

**Figure 3:**
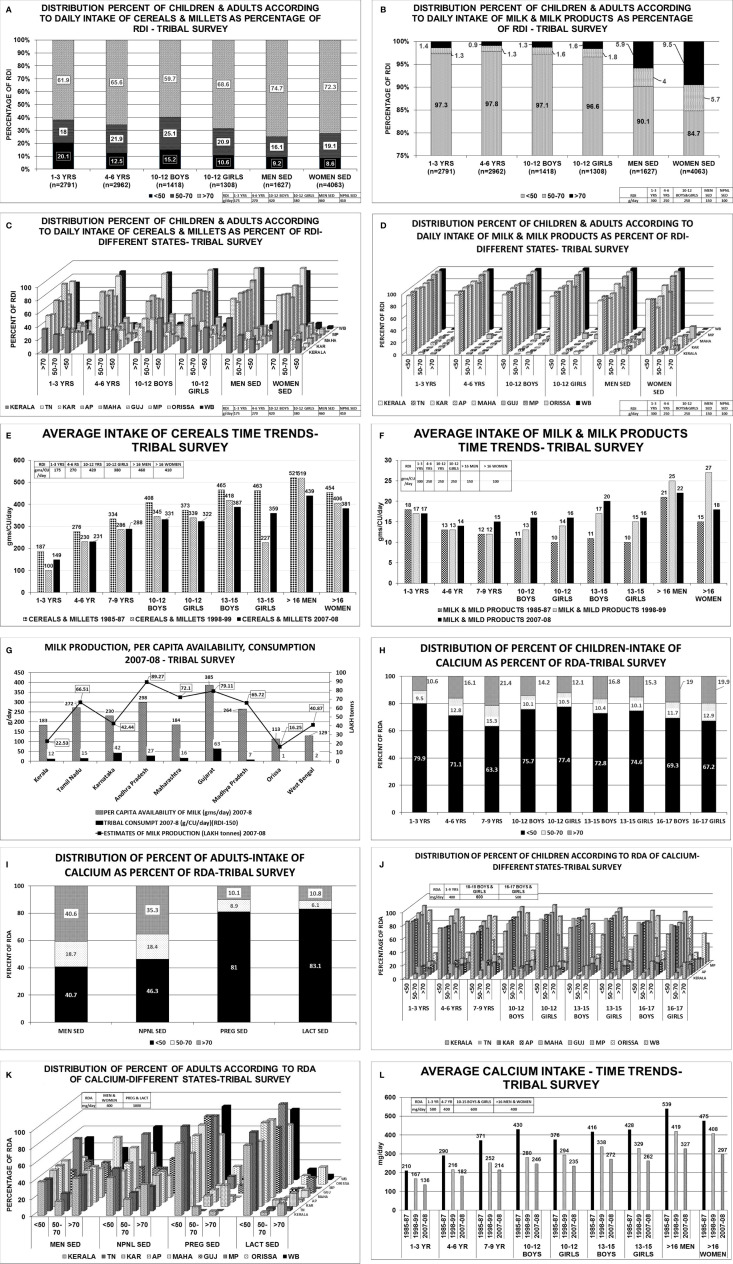
Tribal survey (**Table 1**) (24): **(A)** Distribution percent of children & adults according to daily intake of cereals & millets as percentage of RDI. **(B)** Distribution percent of children & adults according to daily intake of milk & milk products as percentage of RDI. **(C)** Distribution percent of children & adults according to daily intake of cereals & millets as percent of RDI- different states. About 60%–76% of *Children 1–3 years* from AP, Maharashtra, MP, WB, Orissa, consumed >70% of RDI with maximum from Gujarat(82%) and minimum from Kerala(36%). More than 45%–86% of *children 4–6 years* consumed >70% of RDI with exception of Kerala(27%). Among children of *10–12 years boys & girls*, >70% of RDI was consumed from Orissa (88% & 93%) WB (85% & 90%) and least from Kerala(16% & 22%) respectively. In remaining states, consumption ranges from 35% to 68% for boys and 42% to 77% for girls. More than 51%–96% of *adult men* consumed >70% of RDI for cereals & millets from all states. The highest being MP (82%), WB (92%), and Orissa (96%). Similar scenario was seen with non-pregnant women. **(D)** Distribution percent of children & adults according to daily intake of milk & milk products as percent of RDI- different states. Uniformly, 80%–95% of population consumed <50% of RDI of milk & milk products among all age groups in all states. **(E)** Average intake of cereals & millets-Time trends showing a decline in intake of cereals & millets amongst all age groups in all states. **(F)** Average intake of milk & milk products-Time trends showing a decline in intake of milk & milk products across all age groups in all states. **(G)** Graph depicting the milk production and per capita availability for the year 2007–2008. The consumption data of the tribal survey for the year 2007–2008 is superposed. Graph clearly depicts the low consumption of milk despite adequate availability. **(H)** Distribution of percent of children intake of calcium as percent of RDA. **(I)** Distribution of percent of adults’ intake of calcium as percent of RDA. **(J)** Distribution of percent of children according to RDA of calcium in different states. Among *children of 1–3 years* age, about 80%–90% of HH had intake of calcium <50% of RDA and only ~10% of HH had >70% of RDA. Similar scenario was seen in *children 4–6 years* of age. Among *children of 7–9 years* of age, two-thirds of HH had daily intake of calcium <50% of RDA and only 20% of HH had >70% of RDA. Among *10–12 years boys & girls*, 70%–85% of HH consumed <50% of RDA of calcium [except Orissa-43%] and less than 15% of HH achieved the criteria of >70% RDA of calcium[Orissa-37%]. Similar scenario was seen in age group of *13–15 years and 16–17 years boys & girls*. **(K)**. Distribution of percent of adults according to RDA of calcium in different states. Amongst *sedentary men*, 35%–45% of the HH from all the states achieved >70% of RDA of calcium with exception of MP (20%) and Orissa (55%). About 35%–45% of HH of all the states had an RDA of <50% with exception of MP (60%) and Orissa (31%). Similar scenario was seen amongst *non-pregnant, sedentary women*. Only <10% of the HH of *pregnant women* from all states (except karnataka-25%,Orissa 21%) had >70% RDA of calcium. Almost 85%–90% of HH of pregnant women had calcium intake of <50% in all states. Similar scenario was seen amongst lactating women. **(L)** Average calcium intake—time trends showing a decline in dietary calcium intake across all age groups over the past four decades. RDI-Recommended Daily Dietary Intake; RDA-Recommended Daily Dietary Allowance; HH-Households; B-Boys; G-girls; WOMEN NPNL SED-women: nonpregnant, non-lactating and sedentary; WOMEN NPNL MOD-women: non-pregnant non-lactating and moderate; WOMEN PREG SED-women: pregnant and sedentary; WOMEN PREG LACT-women: pregnant and lactating; MEN SED-men: sedentary; MEN MOD-men: moderate; TN-Tamil Nadu; WB-West Bengal; MAHA-Maharashtra; KAR-Karnataka; AP-Andhra Pradesh; GUJ-Gujarat; MP-Madhya Pradesh; UP-Uttar Pradesh.

The consumption of cereals, millets, milk & milk products across all age groups was below the RDI among children, adolescents, men, women-non pregnant, pregnant, lactating, and elderly (**Table 2**; **Figure 3**) (24). The state wise distribution is detailed in **Figures 3C, D**. The milk production per capita availability and consumption for the corresponding period of the survey (2007–2008) is shown in **Figure 3G**.

**Table 2:** Table showing dietary calcium intake of different age groups and various study population across the country from 1999 to 2019.

S.NO	PLACE	AGE YRS	n	STUDY POPU	STUDY TYPE	CALCIUM INTAKE MG/DAY	YR OF STUDY	REF
**1**	PUNE	8.4 ± 1.1	79	VITAMIN D GROUP	VIT D INTERVENTION STUDY	188 ± 56	2019	(26)
**2**	PUNE	7.9 ± 1.2	99	NON VITAMIN D GROUP	VIT D INTERVENTION STUDY	207 ± 54	2019	(26)
**3**	PUNE	8 ± 1.2	192	GOVT PRIMARY SCHOOL BOYS	CROSS SECTI	216 ± 69	2018	(27)
**4**	PUNE	7.9 ± 1.1	167	GOVT PRIMARY SCHOOL GIRLS	CROSS SECTI	194 ± 49	2018	(27)
**5**	BANGALORE	59.05 ± 12.61	252	TERTIARY CARE HOSP	CROSS SECTI	499.94 ± 251.52	2018	(28)
**6**	KOKAN REGION	MEDIAN 14 (MIN-11,MAX-16)	80	ADOLOSCENT SCHOOL GIRLS	CROSS SECTI	MEDIAN-189.4(MIN-49.1,MAX-701.6)	2018	(29)
**7**	MUMBAI	36.50 ± 2.74	265	GROUP-1(NORMAL BMD)SLUM DWELLERS	CROSS SECTI	214 ± 176	2018	(30)
**8**	MUMBAI	37.5 ± 3.44	1135	GROUP-2(LOW BMD) SLUM DWELLERS	CROSS SECTI	301 ± 158	2018	(30)
**9**	PUNE	2–16	220	WHEAT MILK PATTERN DIET	INTERVENTION STUDY	479 ± 222	2017	(31)
**10**	PUNE	2–16	220	RICE PROTEIN PATTERN DIET	INTERVENTION STUDY	351 ± 196	2017	(31)
**11**	PATAN, GUJARAT	12 ± 1.1	30	LOWER SES	CROSS SECTIONAL SEMI URBAN REGION	441.2 ± 227.6	2017	(32)
**12**	PATAN, GUJARAT	11.7 ± 0.5	30	MIDDLE SES	CROSS SECTIONAL SEMI URBAN REGION	484.3 ± 160.9	2017	(32)
**13**	PATAN, GUJARAT	12 ± 1.2	30	UPPER SES	CROSS SECTIONAL SEMI URBAN REGION	749.2 ± 245.4	2017	(32)
**14**	DELHI	28.5 ± 10.40	88	OUTDOOR WORKERS	CROSS SECTI	405 ± 269	2016	(33)
**15**	DELHI	25.8 ± 6.7	32	MIXED GROUP	CROSS SECTI	438 ± 271	2016	(33)
**16**	DELHI	31.7 ± 10.07	74	INDOOR WORKERS	CROSS SECTI	512 ± 228	2016	(33)
**17**	PUNE	27.7 ± 3.5	128	MOTHERS-7 DAYS POSTPARTUM	CROSS SECTI	949 ± 340	2016	(34)
**18**	PUNE	29.4 ± 3.2	88	MOTHERS WITH CHILDREN 1YR AGE	CROSS SECTI	618 ± 256	2016	(34)
**19**	PUNE	29.3 ± 3.0	84	MOTHERS WITH CHILDREN 3YR AGE	CROSS SECTI	530 ± 181	2016	(34)
**20**	BALLABGARH, HARYANA		217	>28-36 WK PREG	CROSS SECTI	858.4 + 377	2016	(35)
**21**	TIRUPATI	40 ± 0.9(MEAN + SEM)	325	RURAL	CROSS SECTI	269 ± 2(MEAN ± SEM)	2015	(36)
**22**	TIRUPATI	47 ± 0.6(MEAN + SEM)	508	URBAN	CROSS SECTI	308 ± 2.3(MEAN + SEM)	2015	(36)
**23**	TIRUPATI	43 ± 0.7(MEAN + SEM)	524	METRO	CROSS SECTI	526 ± 8(MEAN + SEM)	2015	(36)
**24**	VELLORE	58(RANGE:40–74)	106	ERODE TN	CROSS SECTI	632.72 ± 28.23	2015	(37)
**25**	DELHI	23.4 ± 3.9	178	PREGNANT WOMEN	CROSS SECTI	568.0 ± 370.2	2015	(38)
**26**	DELHI	24.7 ± 4.36	158	IMMEDIATE POSTPARTUM WOMEN	CROSS SECTI	634 ± 441	2015	(38)
**27**	MUMBAI	43.21 ± 4.16	76	MALE BMD:Z SCORE>2-NORMAL BMD	COMMU HEALTH CAMP	782 ± 211	2014	(39)
**28**	MUMBAI	39.09 ± 4.02	80	FEMALE BMD : ZSCORE>2-NORMAL BMD	COMMU HEALTH CAMP	590 ± 197	2014	(39)
**29**	MUMBAI	42 ± 4.42	21	MALE BMD:Z SCORE<2-LOW BMD	COMMU HEALTH CAMP	715 ± 201	2014	(39)
**30**	MUMBAI	40.53 ± 4.86	17	FEMALE BMD : ZSCORE<2-LOW BMD	COMMU HEALTH CAMP	514 ± 213	2014	(39)
**31**	ANDHRA (NEAR HYDERABAD)	20.44 ± 1.22	465	VILLAGE WOMEN	CROSS SECTI	423.8(405, 443.5) 95%CI	2014	(40)
**32**	ANDHRA (NEAR HYDERABAD)	20.2 ± 1.2	981	VILLAGE MEN	CROSS SECTI	618.7(600.3, 637.6)95%CI	2014	(40)
**33**	ICDS VILLAGES	9 months-Control group	176	ICDS PROJECT VILLAGES	INTERVENTION STUDY	77(14, 177)-MEDIAN(CI25,75)	2014	(41)
**34**	ICDS VILLAGES	9 months-Complimentary Feeding	177	ICDS PROJECT VILLAGES	INTERVENTION STUDY	127(44, 245)-MEDIAN(CI25,75)	2014	(41)
**35**	ICDS VILLAGES	9 months-Responsive Complementary Feeding and Play Group (RCF&PG)	158	ICDS PROJECT VILLAGES	INTERVENTION STUDY	127(44, 235)-MEDIAN(CI25,75)	2014	(41)
**36**	ACROSS INDIA	41.2 + 10.2-VEGETARIANS	2148	INDIAN MIGRATION STUDY(IMS)	QUESTIONNARIE SURVEY	980.6(751–1247.1)-MEDIAN(IQR)	2014	(42)
**37**	ACROSS INDIA	40.8 + 10.4 NON-VEGETARIANS	4407	INDIAN MIGRATION STUDY(IMS)	QUESTIONNARIE SURVEY	946.5(692.9–1253.1)-MEDIAN(IQR)	2014	(42)
**38**	NORTH INDIA TERTIARY CARE CENTRE	6 MONTHS to 5 YRS	67	CHILDREN WITH RICKETS	RANDOMIZED CONTROLLED TRAIL	204 ± 129	2013	(43)
**39**	KASHMIR	28.75 ± 4.9	64	MEN	CROSS SECTI	368 ± 98.4	2012	(44)
**40**	KASHMIR	26.79 ± 4.71	28	WOMEN	CROSS SECTI	284.4 ± 70.8	2012	(44)
**41**	MUMBAI	25–35	1137	TERITIARY CARE-WESTERN INDIA	CROSS SECTI	322.92 ± 135.17	2011	(45)
**42**	DELHI	18.7 ± 1.2	90	COLL STUDENTS SPORTS GIRLS	CROSS SECTI	779.1 ± 324.5	2011	(46)
**43**	DELHI	NA	96	COLL STUDENTS CONTROLS	CROSS SECTI	409.7 ± 172.5	2011	(46)
**44**	CHANDIGARH	19.4 ± 1.48	329	COLLEGE STUDENTS SUMMER	CROSS SECTI	625.5 ± 273	2011	(47)
**45**	CHANDIGARH	19.4 ± 1.43	237	COLLEGE STUDENTS WINTER	CROSS SECTI	662.6 ± 215	2011	(47)
**46**	PUNE	2.9 ± 0.5 TODDLERS	30	study LOCAL CRECHE OF UNDERPRIVILEG MOTHERS	INTERVENTION	172 ± 82	2011	(48)
**47**	PUNE	2.6 ± 0.5 TODDLERS	28	study LOCAL CRECHE OF UNDERPRIVILEG MOTHERS	INTERVENTION	217 ± 111	2011	(48)
**48**	PUNE	14.1(13.8–14.5)MEDIAN(25%–75%ILE)	100	USES-BOYS	CROSS SECTI	893(689–1295)-MEDIAN(25%–75%ILE)	2010	(49)
**49**	PUNE	14.4(13.8–15.2)MEDIAN(25%–75%ILE)	100	LSES-BOYS	CROSS SECTI	767(585–1043)-MEDIAN(25%–75%ILE)	2010	(49)
**50**	PUNE	14.7(14.4–14.9)MEDIAN(25%–75%ILE)	100	USES-GIRLS	CROSS SECTI	764(541–959)-MEDIAN(25%–75%ILE)	2010	(49)
**51**	PUNE	14.5(14.0–15.1)MEDIAN(25%–75%ILE)	100	LSES-GIRLS	CROSS SECTI	506(380–674)-MEDIAN(25%–75%ILE)	2010	(49)
**52**	DELHI	12.0 ± 2.8	60	LSES-2 MONTHLY GROUP	VIT D SUPPL STUDY	480.8 ± 191.4	2010	(50)
**53**	DELHI	11.4 ± 3.0	64	LSES-MONTHLY GROUP	VIT D SUPPL STUDY	456.3 ± 170.4	2010	(50)
**54**	DELHI	11.6 ± 2.7	81	USES-2 MONTHLY GROUP	VIT D SUPPL STUDY	707.3 ± 162.9	2010	(50)
**55**	DELHI	11.7 ± 2.8	85	USES-MONTHLY GROUP	VIT D SUPPL STUDY	670.5 ± 180.1	2010	(50)
**56**	DELHI	12.4 ± 3.2	193	LSES	CROSS SECTI	454.2 ± 187.4	2008	(51)
**57**	DELHI	12.3 ± 3	211	USES	CROSS SECTI	685.5 ± 184.8	2008	(51)
**58**	DELHI	34 ± 13.1	28	APPARENTLY HEALTHY SUBJECTS	CROSS SECTI	650 ± 409	2008	(52)
**59**	AGOTA VILLAGE,UP	42.8 ± 16.6	32	RURAL MALE	CROSS SECTI	905 ± 409	2008	(53)
**60**	AGOTA VILLAGE,UP	43.4 ± 12.6	25	RURAL FEMALE	CROSS SECTI	595 ± 224	2008	(53)
**61**	TIRUPATI	46 ± 0.43	32	URBAN-MALE	CROSS SECTI	323 ± 8(MEAN + SEM)	2007	(54)
**62**	TIRUPATI	43 ± 1.01	109	RURAL-MALE	CROSS SECTI	271 ± 3(MEAN + SEM)	2007	(54)
**63**	TIRUPATI	46 ± 0.43	476	URBAN-FEMALE	CROSS SECTI	306 ± 2(MEAN + SEM)	2007	(54)
**64**	TIRUPATI	43 ± 1.01	96	RURAL-FEMALE	CROSS SECTI	262 ± 3(MEAN + SEM)	2007	(54)
**65**	PUNE	27.5(IQR 25.6, 29.6)	690	WOMEN PREG 18 WK GA	CROSS SECTI	274-MEDIAN(IQR 223, 354)	2006	(55)
**66**	PUNE	27.5(IQR 25.6, 29.6)	667	WOMEN PREG 28 WK GA	CROSS SECTI	268-MEDIAN(IQR 208, 332)	2006	(55)
**67**	TIRUPATI	59.5 ± 8	164	POST MENOPAU	CROSS SECTI	323 ± 66	2005	(56)
**68**	LUCKNOW	20–29	140	PREG WOMEN URBAN	VILLAGES AROUND	842 ± 459	2005	(57)
**69**	LUCKNOW	20–29	67	PREG WOMEN RURAL	VILLAGES AROUND	549 ± 40	2005	(57)
**70**	BARABANKI DIST,LUCKNOW	14·3 ± 2·7	121	ADOL GIRLS	CROSS SECTI	211 ± 158	2005	(58)
**71**	BARABANKI DIST,LUCKNOW	26·7 ± 4·1	139	PREG	CROSS SECTI	214 ± 150	2005	(58)
**72**	LUCKNOW	6.5 ± 1.2	15	GROUP 1: NOT RECEIVING CAL/VIT D SUPPLI	IDIOPATHIC NEPHROTIC SYNDROME	696.7 ± 73.5	2005	(59)
**73**	LUCKNOW	5.3 ± 0.55	73	GROUP 2:RECEIVING CAL/VIT D SUPPLI	IDIOPATHIC NEPHROTIC SYNDROME	723.3 ± 35.2	2005	(59)
**74**	TIRUPATI	44 ± 1.03(MEAN + SEM)	191	RURAL	CROSS SECTI	264 ± 1.94(MEAN + SEM)	2004	(60)
**75**	TIRUPATI	45.5 ± 0.95(MEAN + SEM)	125	URBAN	CROSS SECTI	356 ± 5.0(MEAN + SEM)	2004	(60)
**76**	LUCKNOW	16.2 ± 2.5	21	SUBJECTS OF OPD	CROSS SECTI	265 ± 199	2003	(61)
**77**	DELHI	22.7 ± 2.8	40	MEN INDIAN PARAMILITARY FORCES	CROSS SECTI	1041 ± 452.6	2003	(62)
**78**	DELHI	23.4 ± 3.1	50	WOMEN INDIAN PARAMILITARY FORCES	CROSS SECTI	764.8 ± 440.2	2003	(62)
**79**	DELHI	25 ± 5 MALE	31	SOLIDER-WINTER	CROSS SECTI	1104 ± 666	2000	(63)
**80**	DELHI	23 ± 5 (M:F-11:8)	19	PHYSICIANS & NURSES-WINTER	CROSS SECTI	879 ± 165	2000	(63)
**81**	DELHI	43 ± 16(M:F-10:5)	15	DEPIGMENTED PERSONS-WINTER	CROSS SECTI	980 ± 300	2000	(63)
**82**	DELHI	24 ± 4(M:F-11:8)	19	PHYSICIANS & NURSES-SUMMER	CROSS SECTI	879 ± 165	2000	(63)
**83**	DELHI	23 ± 3	29	PREG WOMEN-SUMMER-LSES	CROSS SECTI	345 ± 78	2000	(63)
**84**	ALWAR & BHARATPUR RAJASTHAN	6–<12 MONTHS	16		PROSPECTIVE STUDY	514.2 ± 413	1999	(64)
**85**	ALWAR & BHARATPUR RAJASTHAN	12–35 MONTHS	31		PROSPECTIVE STUDY	393 ± 332.5	1999	(64)
**86**	ALWAR & BHARATPUR RAJASTHAN	36–72 MONTHS	13		PROSPECTIVE STUDY	209.7 ± 135	1999	(64)

All values are mean ± SD unless stated. LSES-lower socioeconomic status; USES-upper socioeconomic status; PREG-pregnancy; WK-weeks; GA-gestational age; GOVT-government; BMD-bone mineral density; ADOL-adolescent; SES-socioeconomic status; UP-Uttar Pradesh; ICDS-integrated child development services; SEM-Standard Error Of Mean; IQR-interquartile range; MIN-minimum; MAX- maximum; YRS-years; CROSS SECTI-cross sectional survey; COMMU HEALTH CAMP- community health camp.

### Urban Survey-NNMB 

Cereals and millets (320 g/CU/day) formed major portion of diet in urban population. Less than 70% of HH met the RDI requirement. While 81% of HH met the RDI requirement of intake of milk and milk products, only 67% of HH met the RDA of calcium (25).

### Other Studies

The dietary calcium intake drawn from various studies from 1999–2019 **(** **Table 2** **) (** 26–64) shows intake less than the RDA for various age groups, gender, and physiological states. From a study in South India, the dietary calcium intake of rural, urban, and metropolitan subjects was 268 ± 2,308 ± 2.3, 526 ± 8(mg/day ± SEM)(P<0.001)(RDA-600) (36).

To summarize, the highest intake of cereals and lowest intake of milk & milk products was observed in rural and tribal subjects whereas, the intake of cereals, milk & milk products were similar in both urban and metropolitan subjects. There are similar reports of low dietary calcium intake in postmenopausal women and adolescents migrant workers in metropolitan cities (33, 37, 56, 65). One of the reasons for lower calcium intake was the proportion of calcium derived from dairy sources. As per 1990 Food and Agricultural Organisation (FAO) report, the average per capita calcium intake in developing countries is 344 mg/day compared to 850 mg/day in developed countries.

## Modern India-Historical Changes in Milk and Cereal Production and Consumption Pattern Since Independence

There is a decline in the dietary intake of calcium in the past four and a half decades, as evidenced by NNMB data (1981–2011) in both rural (**Figures 2E, F, K, L** **)** and tribal(**Figures 3E, F, L** **)** population. The average daily dietary calcium intake(from cereals, milk and milk products) across various age groups in both genders, and, pregnant and lactating women were well below the RDA (**Table 1**). Cereals were the only source of calcium, followed by milk and milk products in both rural and urban areas. The quinquennial surveys of the National sample survey organization(NSSO)(68^th^ Round 1987-8 and 2009-10) showed that there is a fall in share of total consumer expenditure for food from 64 to 54% in rural and 56%–47% in urban areas (66).

## Production and Consumption of Milk

White revolution started in India in 1970 (67). India is the leading producer of milk in the world (187.7 Million tonnes in 2018–2019 with per capita availability of 394 g/day;*Source :* National Dairy Development Board) accounting for 22% of global production, with an estimated compounded annual growth rate of ~14.8% between FY 2018 and FY 2023 (68). The milk production and per capita availability of milk in India for the year 1991–1992, 2000–2001, 2010–2011, 2018–2019 are 55.6, 80.6, 121.8, 187.7 million tonnes and 178, 217, 281, 394 g/day respectively (69). Milk and milk products constituted 9.42% and 9.91% (1990–1991), 9.31% and 10.5%(1992), 8.68% and 8.30% (2000–2001), 9.1% and 7.8% (2011–2012) of per capita monthly consumption expenditure of rural and urban subjects (National Sample Survey Office -NSSO 46^th^, 48^th^, 56^th^, and 68^th^ rounds) (69). There are regional differences in milk consumption pattern which may be attributed to lower purchasing power and deviant food habits in the country.

The average consumption of milk & milk products of children and adults from the rural survey of NNMB for 1975–1979, 1988–1990, 1996–1997, 2011–2012 shows about 80%–84% of the children and 56%–62% of adult men and women consumed less than 50% of the RDI(**Figures 2B, D** **) (** 22, 23). Similarly, in the NNMB tribal survey 1985–1987, 1998–1999, 2007–2008 shows 85%–97% of children and adults consumed less than 50% of RDI of milk & milk products (**Figures 3B, D** **) (** 24). Though the consumption of milk and milk products has increased over time, the difference between consumption of various economic strata is quiet glaring. In 1983, the per capita milk consumption by rich households was 8.6, 3.9, 2.2 times of very poor, poor and non-poor households. The gap has reduced to 6.15, 3.5, and 2.05 respectively in the year 2004–2005 and to 6.8, 3.3, and 1.8 respectively, in the year 2009–2010 (70). About 80% of milk production comes from small farmers (69, 71, 72).The per capita consumption of milk is grossly below the per capita availability and the Recommended Dietary Intake (RDI) during the period of respective surveys (**Figures 2G** and **3G**).

## Production and Consumption of Cereals

According to FAO(2010), India is the world’s largest producer as well as largest exporter of millets and 2^nd^ largest producer of wheat, rice, pulses (73, 74). Rice occupies 64.4% of India’s cereal exports (2013–2014) (74, 75). In the past decades, the quantity of *coarse cereals* production has dramatically reduced with increase in production of rice and wheat in food supply. Green revolution transformed India to cereal exporting country. The focus of production was on high yielding rice and wheat. Several government programs were launched—National Food Security Mission (NFSM, 2007–2008) and Bring Green Revolution in Eastern India (BGREI, 2010–2011), which led to increase in production of rice, wheat and cereals (74, 75). There was a change in the consumption pattern with rice and wheat dominating and decline in coarse cereals. Moreover, the Public Distribution System (PDS) allows subsidizing rice and wheat to large percentage of poor households (PDS supplies food grains to 800 million of 1.3 billion population). Proportionally, the total cereal consumption declined from 35% to 5% in rural, 17% to 3% in urban areas in 1961–2012 over the past half a century (74, 76).

Coarse cereals (defined as cereal grains other than wheat & rice) are directly consumed in many developing countries. The nutrient content-kcal, *calcium(mg)*, phytate(mg) per 100 g of edible portion(12% moisture) for various cereals are rice(milled,*oryza sativa*) 356,*33*,266; wheat(atta,*tricium aestivum*) 320,*30*,632; sorghum(jowar;*sorghum vulgare*) 334,*25*,549; pearl millet(bajra;*penniselum glaucum*) 348,*42*,485; maize(*zeus maize*) 334,*26*,646; finger millet(ragi;*eleusine coracana*) 320,*350*,306 respectively (74–79). Finger millets contain highest amount of calcium but with high amount of antinutrient-phytates. Phytates inhibits the micronutrient absorption(calcium) and reduce the bioavailability.

Majority of the Indian population use food grains as the main component of their diet, although there are variations in diet found across different parts of the country. The principal food grain in the North India is wheat followed by rice. In the Western India, wheat comprises of 50% of total grain consumption followed equally by coarse cereals & rice together. In South India, rice is consumed as main food grain. Wheat is the staple food across India consumed using whole wheat flour(atta) in the form of unleavened flat bread(roti,chapattis) and as bakery products. Approximately, 47% urban and 57% of rural subjects’ energy intake comes from cereals for low income populations who obtain high proportion of nutrients and calories from cereals. Cereals contribute to 1 kg/per person/per month in rural India, and 0.8 kg/per person/per month(other than rice and wheat) in urban India (2011–2012) (74).

In 1961–1962, the average 30-day consumption(kg/30 day/person) for cereals in rural subjects in India for rice 8.8, wheat 2.6 and coarse cereals 6.1 which constitute 50%, 15%, 35% of the total consumption respectively. In 2011–2012, it was 6, 4.3, 0.6, constituting 53%, 38%, 5.2% of total consumption respectively. In the urban population, in 1961–1962, the average 30-day consumption(kg/30 day/person) for rice 6.2, wheat 4.1,coarse cereals 2.1 which constitute 50%, 33.2%, 17% of total consumption. In the year 2011–2012, the corresponding values were 4.5, 4, 0.2 constituting 48.4%, 43.2%, 2.6% of total consumption. Hence, for a period of half a century, the 30-day *total* cereal consumption decreased from 17.6 to 10.8 in rural subjects and 12.3 to 8.6 in urban subjects despite ample availability (74, 80–84).

## Changing Rural Urban Consumption Patterns

As per the NSSO data, the Per Capita Cereal Consumption(PCCC) has been declining despite a sustained raise in Monthly Per Capita Consumption Expenditure (MPCE) across the country among both urban and rural households (85).The PCCC depends on region, food habits, education and occupation; and gives consumption data during the preceding 30 days which is a sum of wheat, rice and other cereals. The PCCC (kg/month) in rural India declined from 15.53 (1970–1991) to 11.76 (2007–2008). When the PCCC for each MPCE class are examined, there was a positive and monotonic relation between PCCC and MPCE, the well-off households consumed more cereals than poorer households (in terms of MPCE) (85). In the past decade, the PCCC has declined particularly among higher MPCE classes. The sedentary workers consumed less cereals compared to manual laborers because cereals acted as inexpensive calories for low wage-heavy labor workers with high calorie requirement.

With increasing monthly per capita expenditure (MPCE), there has been a progressive fall in cereal intake. In the urban population, 29% of top and 69% of bottom 5% of population consumed cereals as ranked by MPCE. In the rural population, the cereal consumption was 42% in top and 70% in bottom 5% of population (66, 85). As per the NSSO 68^th^ Round, the consumption of cereal was the highest in the lowest income group, despite spending smaller portions of their income, because the Public Distribution System (PDS) supplied cereals (66, 85). In the middle- and high-income groups, the decline in cereal consumption has been mainly due to consuming fast foods and diet diversification.

## Trends in Consumption and Expenditure

For nearly half a century, the Private Final Consumption Expenditure (PFCE) as percentage of GDP had declined from 79% (1980–1981) to 58% (2014–2015) (66, 85, 86). The PFCE on food items as percent of total PFCE had declined from 61% to 36% and on non-food items has increased from 39% to 64%, between 1972–1973(NSSO 27^th^ round) and 2011–2012 (NSSO 68^th^ round) (66, 87–92). For the corresponding period, the share of expenditure on total food items declined from 72.9% to 48.6% in the rural areas and from 64.5% to 38.5% in urban areas. The expenditure on non-food items increased 27% to 51.4% in rural areas and from 35.5% to 61.5% in urban areas (87–92). Similarly, the share of expenditure on consumption of cereals declined from 57% to 25% in rural areas and 37% to 19% in urban areas. Whereas, the share of expenditure on milk & milk products increased from 10% to 19% in rural areas and 14% to 20% in urban areas (87–92).

## Importance of Cereal as a Source of Calcium in India

In India, the consumption of home-based cereal-pulse vegetarian diets and low intake of dairy products is common. Furthermore, availability and consumption of packaged fortified foods is negligible among Indians. Populations in developing countries, however, have been found to subsist on much lower calcium intakes (~500 mg/day) (31, 93). The main reasons responsible for these differences in calcium requirements are pronounced differences in the intakes of animal protein and sodium between the Indian and Western populations. The animal protein intake in India is far less than in the West (93, 94). Furthermore, in developing countries, non-dairy sources form the major source of calcium intake, especially in India (94, 96). Only 14.3% of urban and 8.7% of rural population consumes the recommended intake of milk and milk products (94).

## Interactions of Vitamin D and Calcium Deficiencies

About 80%–85% of population from India are suffering from various degrees of vitamin D deficiency (97). Vitamin D deficiency in India was documented for the first time while studying a cohort of patients for the cause of bone disease in primary hyperparathyroidism and their control subject (98). Later, other reports documenting with low 25(OH)D levels in children, adults, pregnant and lactating women, adults and elderly subjects ensued (99). The 25(OH)D levels in South Indian subjects are relatively higher than those residing in the north because of equatorial proximity (99). There are *in vitro* studies done at Tirupati, South India (Latitude13.24°) to show adequate vitamin D synthesis on exposure to sunlight (99). In India, the dietary habits vary with different socioeconomic classes and culture across different regions. With mechanization, the lifestyle in modern India has changed to less labor intensive work and prolonged indoor working hours limiting sun exposure which is further amplified by the dress code, use of skin creams with SPF>15 which could account for vitamin D deficiency.

In India, there is a combination of dietary calcium deficiency and vitamin D deficiency (despite adequate sunlight). The secondary hyperparathyroidism (SHPT) which sets in, because of dietary calcium deficiency(declining dietary cereal and milk intake in India) accelerates the conversion of 25(OH)D to 1,25(OH)2D, thereby reducing the serum 25(OH)D concentrations (**Figure 1**). Thus, there is *primary* calcium and vitamin D deficiency and *secondary* vitamin D deficiency due to calcium deprivation (18, 100). Growing skeleton requires calcium requirements to be met. In the background of low dietary calcium intake and poor vitamin D status, the problem is compounded. Similar scenario is encountered in a pregnant, lactating women to meet the demands of the fetus and new born. The intake of milk and milk products coupled with cereals containing high phytate content(ragi), the calcium deficiency is accelerated.

## Impact and Magnitude of the Problem

The combination of changing dietary habits, urbanization and changing lifestyle, magnify the calcium and vitamin D deficiency. Calcitriol supplies the minerals calcium and phosphate, essential for bone stiffness by increasing the intestinal absorption. Calcitriol cannot do this job with low dietary calcium supply, ultimately leading to SHPT causing structural damage to bone—rickets/osteomalacia.

Vitamin D plays an important role in absorption of dietary calcium when its bioavailability is low. When calcium intake is high, passive paracellular calcium transport plays a major role. When the calcium intake is low, vitamin D dependant intestinal calcium absorption is mainly transcellular and dependant on active transport involving calmodulin D_9k_ and vitamin D dependant TRPV6 (2). Approximately, 1/3 of dietary calcium intake is absorbed in vitamin D replete individuals with adequate calcium intake. This is reduced to 10%–15% in vitamin D deficient individuals. Body adapts to the low intake of calcium by reducing the calcium excretion and increasing the fractional intestinal absorption.

From the nutritional data available for past half a century, the dietary calcium intake of infants and children, adults, pregnant and lactating mothers are far below the RDA (**Tables 1** **–** **3**). The peak bone mass is achieved in early adulthood, second and third decade of life tracks through the childhood and adolescence. With advancing age, the bone mass declines and is accelerated at menopause. In a population where there is uniform dietary calcium and vitamin D deficiency across all age groups and in both genders (**Tables 1**, **2**), the peak bone mass achieved is lower compared to the West.

**Table 3:** (104, 105) The daily intake of various food categories in grams per capita per day and nutrients in grams per consumer unit in different states in India[Precision is > 0.05 happens due to low coverage/sample size or high coefficient of variation (SD/Mean) or both].

		CALCIUM	MILK CONSUP	ALL CEREALS	RICE	WHEAT	RAGI & ITS PRODUCTS	JOWAR & ITS PRODUCTS	BAJRA & ITS PRODUCTS	MAIZE & ITS PRODUCTS
	**STATE**	mean ± SD (median) *mg/CU/day*	mean ± SD (median) *g/CU/day*	mean ± SD (median) *g/CU/day*	mean ± SD (median) *g/CU/day*	mean ± SD (median) *g/CU/day*	mean ± SD *g/CU/day* [preci]	mean ± SD *g/CU/day* [preci]	mean ± SD *g/CU/day* [preci]	mean ± SD *g/CU/day* [preci]
1	Andaman & Nicobar Island	484 ± 242(428)	73 ± 119(10)	396 ± 112(395)	322 ± 121(320)	56 ± 43(59)	0	0	0	0
2	Andhra Pradesh	463 ± 202(421)	175 ± 125(147)	443 ± 121(438)	401 ± 120(399)	12 ± 16(9)	7.127 ± 19.954[0]	5.518 ± 18.609[1]	0.268 ± 4.634[1]	0.043 ± 0.782[1]
3	Arunachal Pradesh	258 ± 170(217)	48 ± 99(3)	497 ± 208(468)	441 ± 196(418)	17 ± 35(0)	0.016 ± 1.445[1]	0.167 ± 1.846[1]	0.034 ± 1.678[1]	11.795 ± 36.575[1]
4	Assam	250 ± 125(224)	60 ± 67(46)	483 ± 114(468)	446 ± 118(436)	21 ± 37(13)	0	0	0	0.036 ± 0.953[1]
5	Bihar	424 ± 187(392)	168 ± 130(149)	489 ± 121(474)	244 ± 74(233)	224 ± 70(217)	0.006 ± 0.859[1]	0.000 ± 0.066[1]	0.176 ± 2.716[1]	4.202 ± 15.852[1]
6	Chandigarh	682 ± 302(620)	368 ± 213(314)	296 ± 94(288)	69 ± 53(49)	218 ± 78(215)	0	0	0	0.314 ± 2.814[1]
7	Chhattisgarh	254 ± 156(212)	48 ± 96(0)	489 ± 122(476)	433 ± 132(432)	46 ± 53(34)	0.002 ± 0.138[1]	0.063 ± 0.839[1]	0	1.540 ± 8.685[1]
8	Dadra & Nagar Havelli	348 ± 254(238)	111 ± 166(49)	324 ± 85(333)	226 ± 122(272)	71 ± 98(11)	4.616 ± 15.636[1]	2.978 ± 13.924[1]	2.243 ± 12.581[1]	0
9	Daman & Diu	486 ± 154(448)	168 ± 105(143)	311 ± 73(314)	148 ± 63(144)	101 ± 81(85)	0	18.663 ± 38.160[1]	15.583 ± 29.470[1]	0
10	Goa	453 ± 206(428)	206 ± 138(201)	341 ± 76(332)	250 ± 63(247)	57 ± 40(51)	0.477 ± 3.631[1]	2.518 ± 14.646[1]	0	0
11	Gujarat	556 ± 231(524)	254 ± 164(226)	334 ± 101(331)	77 ± 63(59)	162 ± 88(167)	1.072 ± 11.538[1]	8.042 ± 30.422[1]	50.942 ± 84.274[0]	24.374 ± 72.419[1]
12	Haryana	**890 ± 506(787)**	**533 ± 405(445)**	357 ± 92(351)	34 ± 37(24)	315 ± 90(310)	0	0.014 ± 0.486[1]	3.140 ± 16.877[1]	0.485 ± 3.476[1]
13	Himachal Pradesh	717 ± 374(633)	388 ± 295(313)	472 ± 93(468)	175 ± 69(176)	258 ± 70(257)	0	0.029 ± 2.063[1]	0.265 ± 5.909[1]	**31.523 ± 53.007[0]**
14	Jammu & Kashmir	640 ± 282(571)	351 ± 198(312)	492 ± 125(467)	314 ± 169(286)	133 ± 101(126)	0	0.108 ± 3.115[1]	0.040 ± 1.915[1]	25.336 ± 56.174[0]
15	Jharkhand	344 ± 219(270)	110 ± 145(37)	493 ± 128(491)	343 ± 135(324)	131 ± 96(132)	0	0.035 ± 1.520[1]	0.011 ± 0.456[1]	3.471 ± 15.881[1]
16	Karnataka	577 ± 307(500)	170 ± 117(143)	375 ± 105(369)	221 ± 90(211)	40 ± 31(33)	**39.826 ± 60.075[0]**	**43.563 ± 57.874[0]**	2.417 ± 22.504[1]	0.510 ± 4.793[1]
17	Kerala	439 ± 220(392)	132 ± 128(109)	357 ± 107(347)	284 ± 89(278)	31 ± 27(26)	0.296 ± 2.045[1]	0.026 ± 0.970[1]	0.002 ± 0.091[1]	0.021 ± 0.760[1]
18	Lakshadweep	346 ± 133(328)	16 ± 29(10)	408 ± 111(418)	331 ± 99(330)	32 ± 24(30)	0	0	0	0
19	Madhya Pradesh	456 ± 239(410)	180 ± 166(142)	459 ± 130(448)	86 ± 94(44)	342 ± 147(338)	0.001 ± 0.057[1]	5.024 ± 41.985[1]	0.147 ± 11.277[1]	16.951 ± 66.779[1]
20	Maharashtra	453 ± 200(423)	165 ± 129(141)	372 ± 109(374)	123 ± 82(105)	169 ± 84(164)	1.116 ± 12.113[1]	38.941 ± 62.621[0]	15.007 ± 40.387[0]	0.496 ± 6.969[1]
21	Manipur	183 ± 76(167)	18 ± 38(3)	**554 ± 105(549)**	**549 ± 106(543)**	1 ± 10(0)	0	0	0	0.044 ± 0.661[1]
22	Meghalaya	235 ± 114(221)	52 ± 61(34)	405 ± 64(400)	377 ± 65(375)	9 ± 17(0)	0	0	0	1.075 ± 5.976[1]
23	Mizoram	273 ± 158(215)	55 ± 83(8)	500 ± 121(489)	484 ± 118(476)	4 ± 25(0)	0	0	0.008 ± 0.355[1]	3.224 ± 15.081[1]
24	Nagaland	217 ± 90(206)	20 ± 40(11)	491 ± 83(491)	476 ± 88(482)	3 ± 21(0)	0	0	0	3.552 ± 16.885[1]
25	NCT of Delhi	651 ± 262(615)	329 ± 178(298)	305 ± 92(304)	76 ± 43(70)	214 ± 82(214)	0	0	0.014 ± 0.424[1]	0.329 ± 2.606[1]
26	Puducherry	590 ± 220(569)	246 ± 139(242)	368 ± 109(360)	309 ± 100(300)	36 ± 25(36)	1.768 ± 6.043[1]	0	0.009 ± 0.270[1]	0.009 ± 0.207[1]
27	Punjab	823 ± 367(753)	469 ± 282(412)	353 ± 83(346)	37 ± 39(27)	307 ± 83(301)	0	0.006 ± 0.347[1]	0.028 ± 0.493[1]	4.394 ± 13.683[1]
28	Rajasthan	713 ± 355(641)	380 ± 275(312)	460 ± 112(449)	12 ± 17(8)	**367 ± 149(381)**	0.001 ± 0.067[1]	0.890 ± 18.567[1]	**65.023 ± 138.489[0]**	11.463 ± 47.453[1]
29	Sikkim	497 ± 183(485)	255 ± 138(258)	408 ± 96(406)	354 ± 92(356)	25 ± 21(22)	0	0	0	6.852 ± 17.477[1]
30	Tamil Nadu	466 ± 202(433)	181 ± 128(157)	359 ± 102(352)	320 ± 103(313)	23 ± 24(19)	3.568 ± 12.820[0]	0.146 ± 2.540[1]	0.151 ± 2.509[1]	0.081 ± 1.499[1]
31	Telangana	407 ± 171(382)	155 ± 106(134)	451 ± 115(444)	405 ± 115(401)	21 ± 25(15)	0.358 ± 2.501[1]	12.509 ± 35.192[1]	0.011 ± 0.417[1]	0.138 ± 2.076[1]
32	Tripura	291 ± 131(257)	49 ± 80(3)	548 ± 108(540)	521 ± 106(515)	8 ± 15(0)	0	0	0	0.138 ± 2.358[1]
33	Uttar Pradesh	501 ± 286(427)	220 ± 215(168)	450 ± 116(442)	155 ± 97(159)	285 ± 102(269)	0	0.131 ± 4.972[1]	1.354 ± 12.830[1]	0.894 ± 7.937[1]
34	Uttarakhand	649 ± 296(598)	308 ± 208(269)	481 ± 106(474)	190 ± 74(183)	276 ± 77(272)	4.033 ± 13.791[1]	0.002 ± 0.167[1]	0	0.432 ± 5.292[1]
35	West Bengal	321 ± 163(282)	72 ± 95(39)	450 ± 122(442)	354 ± 134(349)	66 ± 56(54)	0.000 ± 0.189[1]	0.000 ± 0.024[0]	0.000 ± 0.031[0]	0.070 ± 1.640[1]
36	Odisha	282 ± 155(237)	60 ± 94(5)	526 ± 124(519)	468 ± 137(465)	33 ± 45(16)	1.578 ± 8.559[1]	0.040 ± 1.299[1]	0.050 ± 2.295[1]	0.401 ± 4.931[1]

Bold values indicate- highest daily intake of various food categories in different states.

Globally, 800 million people are undernourished. More than 3.5 billion people are at risk of calcium deficiency and 90% of those at risk, are in Africa and Asia (101). In one of the longest (1963–2005) and largest study from India, nutritional bone disease constituted 52% of the population (102). The commonest disorders are rickets (7.6%), osteomalacia (35.3%), and osteoporosis due vitamin D deficiency, because of inadequate sunlight exposure and dietary calcium deficiency(<300 mg/day) (102). In India, osteoporotic fractures occur in both males and females a decade earlier (103). There is good evidence to show that low dietary calcium intake of 200 mg/day is associated with impaired mineralization and rickets.

ICMR-NIN TATA center dashboard (104) provides information on intake of different macronutrients and micronutrients by age and gender population categories using the latest Indian Food Composition Tables to estimate nutrient content of raw food items (77). A total of 101,204 households comprising of 463,622 members of all age groups, both genders of rural and urban were evaluated across the country to derive population category estimates(Consumer Units). The daily dietary intake of various food categories in grams per capita per day and nutrients in grams per consumer unit in different states in India are shown in **Table 3**.The table shows the diversity of cereal intake across different states in India. The total daily intake of calcium(mg/cu/day) in India is 462 ± 179(rural-447 ± 192, urban-493 ± 155), is far below the RDA (77, 104).

## RDA and Recent Revisions

The ICMR-NIN (105) Recommended Dietary Allowances(RDA)(mg/day) 2020 for various age groups as follows(values from USA-Institute Of Medicine-IOM 2011 is given in brackets): 0–1 year:300(200–260); 1–3 years:500(700); 4–6years:550(1000); 7–9 years 650(1000–1300);10–12 years:850(1300); 13–15 years:1000(1300); 16–18 years:1050(1300);>18 years:1000(1000); >65+: NA(1200); pregnant women 1000(1300) and lactating:1200(1300). The values recommended by FAO and USA-IOM are similar (106, 107). The RDA has undergone upward revision(since 1979) and is still not at par with RDA of West. The revised ICMR-NIN guidelines are well intended but needs some updates (108). Added to this, there is uniform vitamin D deficiency in Indian population as well.

To summarize, though India is the largest producer of milk and cereals, the major source of calcium in India is through non-dairy products. With modernization the PCCC has declined in population with higher MPCE. The lowest income group gets the cereals from the PDS. In the middle and high income group there is a decline in the cereal consumption due to consumption of fast food and diet diversification. Strengthening cereal intake and encouraging dairy products goes a long way in addressing the problem of dietary calcium deficiency in India.

## Strategies to Combat Dietary Calcium Deficiency—the Way Forward

In India, there is dietary calcium deficiency across various age groups and gender coupled with vitamin D deficiency. Addressing both simultaneously, is most important (109). The main source of dietary calcium across the population in India has been from non-dairy sources. India should focus on multi-pronged strategy with three main focus areas to tackle the problem of strong bone health while solving the problem of nutritional deficiency. *Firstly*, while the government has many initiatives targeted to achieve nutrition sufficiency, the impact of these are yet to be realized and more emphasis is needed on strong bone health. *Secondly*, corporate India needs to focus on bone health as a part of Corporate Social Responsibility (CSR), along with government and non-profit organizations. *Lastly*, as we are living in a digital world, it becomes imperative to leverage technology to solve societal problems and realize the impact of initiatives targeting nutrition sufficiency through innovative approaches.

## Governmental Initiatives

India has plethora of polices and nutrition programs to address several decades of malnutrition, but there is paucity of impact. The Indian social safety net program(SSNP) (110) is a platform to address three major nutrition and feeding programs: a) The Integrated Child Development Scheme(ICDS) by the Ministry of Women and Child Development(MOWCD) providing food supplements at community-level, *Anganwadi* centers addressing the need of pregnant and lactating women and children under 6 years of age, b) Mid–Day–Meals scheme(MDM) catering primary and upper primary school children, and, c) Targeted Public Distribution System(TPDS) supplying food grains to three target populations namely, 1) Below Poverty Line (BPL) (to the poorest families), 2) *Anatyodaya Anna Yojana* (AAY) (poorest 20% BPL), and, 3) Above poverty line (APL) (low income not among the poorest **(** **Table 4A** **)**. Apart from these, there are micronutrient supplementation programs and Food Fortification programs. There has not been much emphasis on calcium as a supplement, in micronutrient supplementation or food fortification programs in India (110, 111).

- *Integrated Child Development Services (ICDS)* is the world largest community based program launched in 1975. It is an excellent program for mother and child development. It is a symbol of India’s commitment to her children (111, 112) **(** **Table 4A** **).**
- *National Guidelines for Calcium Supplementation during Pregnancy and Lactation* by Maternal Health Division of Ministry of Health & Family Welfare, Government of India, advocates supplementation of calcium to all pregnant women (113). However, the dose of vitamin D advocated is far less keeping in view of the widespread vitamin D deficiency, and the endocrine society guidelines for treatment vitamin D deficiency for population at risk **(** **Table 4A** **)** (114, 115). The government can mandate inclusion of RDA percentage along with the nutrient information provided in packaged food items to increase awareness and to ensure nutrient intake in both rural and urban India.
- *Mid–Day–Meals Scheme (MDM)*: In the year 1925, MDM was introduced in Madras Corporation for disadvantageous children (120, 121). The Special Nutrition Program (SNP) and Applied Nutrition Program(ANP) was started in the years 1970 and 1973. In the year 1975, Tamil Nadu, Gujarat, Kerala, and Pondicherry universalized the cooked MDM for children at primary school stage with the concept “an undernourished and hungry child(classroom hunger) can never be attentive in class room”. Realizing this relationship, the National Program of Nutrition Support to Primary Education (NP-NSPE) or MDM was universalized for the whole country in the year 1995. This scheme was supposed to promote the enrolment, attendance, and retention of students apart from enhancing gender and caste parity and fostering social equality to those belonging to disadvantaged group. Today, it is the world’s largest school meal program.

**Table 4A:** Table showing strategies to combat dietary calcium deficiency governmental and digital in rural and urban region.

S.NO	Program	TARGET POPULATION	FEATURES	IMPACT	LIMITATIONS	REMEDIAL MEASURES
1	**Indian Social Security Net Program (SSNP)**					
	a) Integrated Child Development program (ICDS)	Community level 0–6 years age	Food supplement at community level	Reduced school dropouts	Did not help achieving nutrition sufficiency 1) The food became the only source of food rather than a supplement. 2) The nutrition composition did not give much weightage to dietary calcium. 3) Children did not attend at very early age of 0–3years, before malnutrition sets in (111, 112)	Instant or precooked fortified products for infants and children can be given: complementary food supplements, micronutrient powders(can be used as home fortificants or point-of-use fortificants) and fortified blended foods. Cookies, biscuits, compressed bars and chikkies are other type of fortified complimentary foods that can be used.
	b) National Guidelines for Calcium Supplementation during Pregnancy and Lactation	Pregnant women from 1^st^ trimester till 6 months post-partum	500 mg elemental calcium and 250 IU Vitamin D_3_ *twice a day*	Increase in awareness of nutrition during and after pregnancy	Calcium and Vitamin D dose advocated is far less than the guidelines for treatment deficiency for population at risk (113–115).	1) Upgrade vitamin D and calcium dose. 2) Continue supplements till 2 years postpartum 3) Compliment feeding with multiple fortifications of essential micronutrients
	c) Mid–Day–Meals Scheme (MDM)	1) Primary stage (class 1–5). 2) Upper primary stage (class 6–8)	Nutrition norm: 1) primary stage - 450 calories. 2) upper primary stage-700 calories	Increase in school going children with 25 crore children studying in 15 lakh schools	1) In year 2018–2019, there was 25% gap in coverage (116). 2) Bridges the food gap, but not the nutrition gap. 3) Little emphasis on calcium and vitamin D	1) Educate parent and children. 2) Supplement fortified milk(with calcium and vitamin D) at the beginning and end of school session–preferably in tetra packs–to prevent adulteration, dilution and pilferage. 3) Inclusion of cheese and paneer in MDM. 4) Supply of fortified flour (with calcium and vitamin D) instead of grains used for preparing MDM. 5) Encourage schools to grow vegetables rich in calcium in kitchen garden for MDM. 6) Increase in adoption of MDM by charitable institutions and CSR if required. 7) Distribution of snacks with high calcium content to children as mid-morning and evening snack for all age groups (117, 118). 8) Snacks made of Gingelly(Sesame) seeds rich in calcium. 9) poster and verbal education at PDS centers
	d) Targeted public distribution system	1) Below Poverty Line(BPL)(to the poorest families), 2) *Anatyodaya Anna Yojana* (AAY)(poorest 20% BPL), and, 3) Above poverty line(APL)(low income not among the poorest				
	*1)Targeted fortification*	1) Lower socio economic strata 2) Those on Public Distribution System(PDS) 3) MDM/ICDS				1) Involve ISKON, Akshaya Patra, 2) fortified ready to eat snacks
	*2) Mass fortification*	Discussed in our previous review (109).				
	*3) Market driven fortification*	Growing children, pregnant and lactating mothers, health conscious population, Subjects recovering from chronic illness,				Fortification of milk, flour, salt oil etc.
2.	**Food fortification**			It can deliver significant proportion of RDA for a number of micro nutrients without necessitating a change in dietary pattern of the population on a continuous basis and without calling for individual compliance	Cannot correct all or either of severity of micro nutrient deficiency, locality or poverty limiting the access of the FF	This problem is overcome by the PDS which caters to 80 crore population *(vide supra).*
	a) Home fortification (HF)	At Household level	Encourages self-reliance, distributes cost effectively and widely, allows freedom of individual choice to utilize the additives		1) No guarantee the target population would participate, 2) The supply of additives has to be replenished to sustain HF, 3) Uncomfortable feel of adding a substance to their food without knowing what it is and 4) The barrier of using additives to regular cooked foods.	Overcome by educational programs (119).
	b) Commercial and industrial fortification (IF)	reaches large populations through PDS or retail stores	Can be made available at low costs with high quantity of production		The producer may drastically increase the price or some may unknowingly perceive the addition as unethical practice.	Overcome by legislation (119).
	c) Biofortification (BF) (genetically modified)	can benefit large population	Well suited for daily diet of low income population using large staple foods.	it requires minimal intervention, is highly sustainable once it is introduced,	Improper knowledge may impact the mono cultures and reduce the bioavailability	Overcome by educational programs

*Limitations:* The standard nutrition norm of MDM are: for *primary stage* (class 1–5) is 450 calories, food grains (100 g), pulses (20 g), protein (12 g), vegetables (50 g), oil and fats (5 g) and *upper primary stage* (class 6–8)-700 calories, food grains (150 g), pulses (30 g), protein (20 g), vegetables (75 g), oils and fat (7.5 g). There are many evaluation studies of nutrition analysis of MDM found based on a number of evaluation criteria (122). In a study aimed at improving the nutrition quality of mid-day-meal scheme, baseline nutrient intake data of children showed high prevalence of undernourishment with micronutrient deficiencies(especially iron, calcium and vitamin A). The percentage of children consuming less than 50% RDA of calcium were:7–9 years-43.8%; 10–12 years-Boys-60.6%, Girls-65.2%; 13–15 years Boys 56.9%,Girls-57.8%; and 16–17 years-Boys-49.2%, Girls-48% (123, 124). In another study from Ludhiana district of Punjab, showed that MDM was found to be a substitute rather than supplement for the home meal. The average daily calcium intake by urban and rural school children was 375 ± 79 and 400 ± 128 (mean ± SD) mg/day. The nutritional contribution of MDM was 108 ± 11 and 107 ± 12 (mean ± SD) mg/day respectively. The percentage contribution of MDM to calcium intake was only 27% of required nutrient intake and 17.8% of RDA intake (125). Similar studies from Haryana of 50 girl children (7–10 years), only 51% consumed RDI of milk and milk products (mean ± SD : RDI) (128 ± 80:250) as per ICMR standards and 60% of them met the RDA of calcium (mean ± SD : RDA) (364 ± 153:600) (126). In another study from Tirupati, with MDM in school going boys and girls of 8, 9, and 10 years, the dietary calcium intake (mean ± SD : RDA) of boys was 234 ± 34:400;237 ± 57:400 and 341 ± 53:600 and for girls218 ± 36:400; 215 ± 55:400 and 303 ± 54:600 respectively. Only 39% of children of both genders consumed milk and milk products daily (127, 128) **(** **Table 4A** **)**.

Majority of the states do not supply milk or milk products in the MDM, despite India being the largest milk producer in the world. Milk has been started as a supplement in Chandigarh, Daman & Diu, Delhi, Gujarat, Haryana, Jharkhand, Karnataka, Kerala, Madhya Pradesh, Pondicherry, Rajasthan, Uttarakhand, and West Bengal only for primary stage students with intake varying from one to five times a week.

**Road Ahead:** MDM bridges the food gap, but not the nutrition gap. The focus of nutrient supplementation was aimed at calories, protein, iron and vitamin A with little emphasis on calcium and vitamin D which are essential for bone health. The solutions for improving nutrition intake in children are dealt in **Table 4A**.

**Targeted Public Distribution System:** Targeted, mass and market driven fortification has been extensively dealt with in our previous review (109).

**Food Fortification:** Addition of one or more essential nutrient to food for the purpose of correcting or preventing a demonstrated deficiency in a population is called Food fortification(FF). It differs from enrichment where the lost nutrients during refinement or production is restored. Different types of FF are: home fortification(HF), commercial and industrial fortification(IF), biofortification(BF) (genetically modified), microbial fortification and synthetic biology (119). The pros and cons of each of these types are enumerated in **Table 4A**.

- *Advantages of FF*: It helps maintain body stores which are essential for growing children. For those who don’t like or allergic to dairy products, calcium fortified foods are a good option.
- *Limitations of FF:* When the micronutrient deficiency affects a large proportion of population, the targeted population FF alone, this problem is overcome by the PDS which caters to 80 crore population* (vide supra).*

The *Pros and cons* of HF, IF, and BF dealt in **Table 4A**.

## Importance of Cereals as a Source of Calcium Methods to Enhance Its Bioavailability

Amongst the cereals, finger millets has the richest source of calcium (350 mg/100 g) and highest phytate content (306/100 g). It requires minimal inputs for growth. It is stress resilient and best suited for sustainable agriculture. The calcium content is 10 folds higher than brown rice, wheat or maize and three times more than milk (129). Besides, it contains high content of other nutrients and is called as *“super cereal”.* But the phytate in the cereal chelates calcium in the gut and reduces its bioavailability. The methods to improve the nutritive value and reduce the phytate content include biofortification and genetic improvement, pre-treatment methods as enzymatic treatment with phytase of grains, precooking treatment methods as fermentation and soaking.

There are gene banks across the globe to develop ca-biofortified finger millets (129). Other methods to enhance the bioavailability of calcium and to reduce phytic acid content of the grains are: *a) sprouting* – lowers phytate and improves extractability of calcium after 4 days of germination and reduces phytic acid content to undetectable levels (130), *b) flour* made from whole grains have higher calcium content(325 mg/100 g) compared to decorticated ones (222 mg/100 g), *c) fermentation* reduces the phytic acid levels by 72.3% and 54.3% after 96 and 72 h, and, d) other methods, as co-fermenting with horse gram increases the nutritive value (131–133).

## Comparison of Calcium Salts for Fortification and Bioavailability

The bioavailability of calcium in fortified food varies. Meal load is inversely related to calcium absorption. Solubility of calcium salt is not proportionate to its bioavailability. It has little effect on absorption of calcium *per se*. The solubility and absorption are extremely low for calcium oxalate. On the other end of the spectrum is calcium citrate malate (CCM) and amino acid chelate bisglycinocalcium, with high solubility and fractional absorption. Inhibitors in the food matrix inhibit calcium absorption(oxalic acid found in spinach, sweet potatoes and legumes. Exception – soybeans have high oxalate content and also high calcium bioavailability). Phytates (phytic acid) reduce calcium absorption. The inhibitory effects can be reduced substantially by processing methods which hydrolyze or remove phytates–fermentation, use of *phytase* sprays and selective precipitation of proteins. Dietary fiber is another inhibitor of calcium absorption and the effect is serious when the calcium intake is low. It can be overcome by small increase in dietary calcium (134, 135). Certain digestive products, milk proteins(caseinphosphopeptides) and lysine-an amino acid, enhance calcium absorption.

***Selecting the Calcium Fortificant***: To fortify food the ideal calcium source should be inexpensive, safe, compatible with food delivery vehicle, highly absorbable and should enhance the bone mass. Only CCM and calcium carbonate(CC) have undergone rigors testing in this way. CC is the least expensive salt and is ideal for flour fortification. Thermal processing liberates CO_2_ from CC. But in fruit beverages CCM is very compatible. CC is used where chalky mouth feel and clarity would not be noticed(food bars and cereals), especially soy and meal replacement beverages. In nonclear beverages, calcium phosphate acts as a buffer to control acidity. In clear beverages, calcium citrate is soluble at certain pH, works and with change in concentration and pH the choice moves to calcium lactate, gluconate and gluconate salts with undesirable acceptance with usage over 10% DV. Calcium fumarate has significant slower dissolution and useful for clear beverage for taste. Syrups and slurries are achievable with lactate and lactate gluconate.

Calcium supplementation has significant impact on BMD and BMC. It has been shown in prepubertal girls that it has a positive impact on the stature/height (136). Calcium deficiency along with other micronutrient deficiency may be the cause of stunting in India.

***Barriers to Effective Fortification:*** The focus of FF should be on the quantity added and the quality of fortificant added. This depends on the choice of salt, manufacturing process, taste effects and stability of the product. Quality related issue is bioavailability and the risk of renal stone formation. In the only published RCT, dietary calcium at recommended levels resulted in 50% fewer stones compared to calcium-restricted diet (137). Similar observations are recorded with rise in calcium intake from low to currently recommended levels (138). The explanation being that the digestive residue complexes which contain unabsorbed calcium blocks the dietary oxalates which is a potent risk factor.

Vitamin D status of an individual affects calcium absorption. The intestinal calcium absorption improves by 68% when the 25OD levels are >30 ng/ml (139, 140). Calcium is best absorbed in smaller amounts taken throughout the day several times. The most effective fortification is levels up to 30% DV (Daily value) i.e. 300 mg per serving: a) Foods containing 10% of DV are–calcium enriched or more calcium or calcium fortified, b) 10%–19% of DV– provides calcium or contains calcium or good source of calcium, and c) >20% of DV– rich or high in calcium or excellent source of calcium. Remedial measures of vitamin deficiency is addressed in our previous publication (109).

## Role of Corporate Sectors in Tackling Nutrition Deficiency in India

There are many government initiatives to address nutrition deficiency, however, the impact of these initiatives is yet to be realized. As a part of CSR, the corporate sector has invested to help address the issue of nutrition deficiency in India. To support Indian government’s Poshan Abhiyaan, Tata Trusts, UNICEF, Sight and Life, CSRBOX, CII, WeCan and NASSCOM Foundation launched an initiative - IMPAct4Nutrition. This initiative allows private sectors to build strategies to combat nutrition deficiency and actively engage to build a social movement as well as build assets to convey the importance of nutrition sufficiency (141). This initiative is expected to be adopted by various corporate bodies to work on tackling the problem of nutrition deficiency. While this initiative is in its nascent stage, government needs to create more such opportunities for corporate sector, either through tangible benefits (tax rebate, allowances) or through intangible benefits (awards, accolades), to have a larger impact on nutrition and wellbeing.

National Dairy Development Board (NDDB), through its NDDB Foundation for Nutrition(NFN) has started “gift milk” program wherein the state milk unions or dairy cooperatives provides milk to children in government schools. The NFN mobilizes funds through CSR to run this initiative and is providing milk to about 118 schools across Delhi, Gujarat, Jharkhand, Maharashtra, Tamil Nadu, Telangana (142, 143). Reckitt Benckiser and Apollo Hospital Group have partnered to launch “Arogya Rakshak - Protected by Dettol, Cared by Apollo” initiative which targets health outcomes of people. This initiative is aimed to complement Dettol’s Swasth India program and Apollo’s Total Health program. The two brands are planning to launch a Center of Excellence(CoE) for community nutrition. One of the initiatives for this CoE is to train the frontline health workers through virtual reality and gamification as well as use of artificial intelligence to assist healthcare workers in detecting malnutrition (144). Microsoft has helped develop Child Growth Monitor to help fight malnutrition. Powered by Microsoft’s artificial intelligence technology and Azure services, the app monitors children’s growth and levels of nutrition (145). There have been some initiatives targeting nutrition by various corporates, however, there are few corporates who are focusing on nutrition for strong bone health across children, pregnant women and adults.

Many corporate hospitals have separate nutrition department. However, this segment provides services mostly for patients with co-morbidities to help them manage their diet. There is very minimal focus or no focus on nutrition for children and adults, specifically for bone health. The corporate India can increase its focus on helping India tackle the nutrition deficiency. There are many cases where the corporate India has helped in tackling the problems of hunger and wasting, however, we are yet to see significant impact of corporate India in helping deal with nutrition deficiency. As a motivator for the corporate sector to support strong bone health of children and adults, it will become necessary for the government to provide tangible and intangible benefits for companies that may help realize significant impact on nutrition deficiency.

## E-Health and M-Health Approaches for Adequate Calcium Intake Across Rural and Urban India

Traditionally, delivery of services was based purely on the physical presence of healthcare workers, non-profit organizations and local knowledge among the communities. With the advent of digital technologies, the healthcare workers have been able to deliver services to the rural regions, thereby increasing the penetration of delivery of healthcare across the remote regions. Digitalization of healthcare sector has also led to advancements in add-on services targeted at nutritional intake of individuals across urban sectors. All these mechanisms use technologies such as mobile apps, analytics, artificial intelligence delivered through internet connectivity and are known as e-health and m-health services.

The problems of nutritional deficiency dealt at rural and urban regions remain divergent. The rural India lacks the understanding and benefits of adequate nutrient intake while the urban India understands the importance of nutrient intake but struggles with consistent intake of balanced diet due to dietary and lifestyle habits. The e-health and m-health services help to address these problems and aid in adequate nutrition intake including calcium for strong bone health.

The technological solutions or the strategic approaches that can be taken across rural and urban India are listed in both the **Table 4B**.

**Table 4B:** Table showing strategies to combat dietary calcium deficiency governmental and digital in rural and urban region.

Solutions/Approaches for Rural Regions.	
**Communication through digital channels**	•Educate the community about the nutritious food through broadcast on digital channels. Program aired on Vande Gujarat Channel-1 and streamed through YouTube/Facebook has nutrition experts providing guidance through digital channels (146). This initiative can be extended across multiple channels and programs to expand reach across regions, including Mann-ki-Baat and through smartphones.
	•Deliver educational content in classrooms, availability of educational games in the mobile apps and television programs can shift the attitude towards consuming of nutrient rich food (147).
	•Drawing parallels from the agriculture industry where mobile phones are used as a fast and easy access to communicate and deliver information (148), another proposition is to set-up a framework of telephonic information delivery system wherein the call center can address the queries and concerns regarding the intake of nutritious food and the impact thereof. Since this proposition can be delivered through the voice calls and does not require internet availability, it will help expand accessibility to remote regions and increase awareness of nutritious foods.
**Increase awareness**	•Information about importance of nutrition rich food through educational toys for children, targeted advertisements by the local community and local governance bodies helping to increase awareness and availability of calcium rich foods.
	•To address the problem of digital exclusion among marginalized communities and to educate them of importance of calcium rich diet, verbal education about importance of strong bone health can be delivered through local nutrition experts or Anganwadi workers. This can also be taken up at the public distribution centers for cereals and grains.
**Aadhar-based milk banks**	•Aadhar-based milk banks can also be set-up, wherein IoT-based scanners can be used to scan card and provide milk supply twice a day for each person which will need to be consumed immediately.
**Solutions/Approaches for Urban Regions**	
**Use of mobile apps**	•Technology solutions act as a guidance for self-management of adequate intake of nutrient rich food including calcium for bone health. There are many mobile apps available to track the calcium intake in the daily diet. The mobile apps inputs information of the user’s daily intake of calcium through recall, which is used to estimate the gap between daily intake and RDA, thereby suggesting the additional intake required based on RDI. These apps provide basic information and needs to be built for a holistic approach of providing personalized service including periodic monitoring.
**Use of analytics and AI**	•There are many apps available based on analytics and artificial intelligence (AI) to help users maintain nutritious and balanced diet. These apps use predictive analytics, artificial intelligence and natural language processing to help track and monitor nutrition intake (149). These apps are mostly marketed as lifestyle requirements rather than necessities. Most of these apps do not allow monitoring of historical data of user which can help devise long-term goal setting and suggest behavior changes in lifestyle (150).
	•Nestle India has launched artificial intelligence (AI) based nutrition assistant with a voice-activated functionality which responds to nutrition queries. •Nestlé India Nutrition Assistant (NINA), built in association with Google, focuses on parents and caregivers of children up to 12 years of age and suggests custom meal plans personalized to user preferences. AI fuels automated learning system in the app and drives self-learning as the usage of the app increases (151). The adoption of this assistant is still in the beginning phase and additional emphasis on calcium intake will be required for children. This app can be used to across India in local vernacular languages and should be made accessible through a mobile app.
**Increase awareness**	•In India, there is a need for industry endorsed calcium management apps which allows self-tracking of calcium intake through periodic recording of food and nutrient intake as well as risk assessment.
	•There are many computer-based tools available for guided dietary intake of nutrition which can also be used for bringing the awareness and importance of calcium intake for bone health. It has been proven that computer-based self-management tools for calcium intake has led to higher level of calcium intake over a period of time (152).

While there are myriad of solutions available to tackle the deficient intake of nutrition in both urban and rural India, they are not utilized to its full potential. Innovative ideas need to be leveraged to improve the awareness and impact of nutrition sufficient diet in both rural and urban India to achieve the target of Swasth Bharat.

## Conclusion

Despite India being the largest milk and cereal producer in the world, there is widespread dietary calcium deficiency in India in the background of vitamin D deficiency. This indicates a gap between production and consumption in Indian dietary pattern with reference to dietary calcium. In the “*production-supply-consumption chain*”, India needs to introspect and adopt measures to increase the dairy and non-dairy sources of calcium which can address various segments of population with diverse cultural habits. Correction of dietary calcium deficiency will also partially correct secondary vitamin D deficiency. This can be achieved by targeting through the Indian Social Safety Net Program (SSNP) and also using technology to address this issue. The existing measures like MDM are viewed by beneficiaries as “food substitute” rather than as “food supplement” which was the concept while introducing the program—this misconception needs to be addressed. On the part of the government they should aim at bridging the “nutrient gap” and not the “food gap” in the MDM. The government should consider a legislation for fortification of food with calcium and to bring the calcium supplements under price control act to make it affordable for the common people. Till these programs are implemented, it is prudent on the part of people to meet the RDA of calcium either by choosing their dietary pattern to be calcium sufficient or by supplementing calcium to meet the goals (109, 116, 117). India needs to introspect and to plan in prospect the way ahead to combat this dietary calcium deficiency.

## Search Strategy and Selection Criteria 

References for this review were identified through searches of PubMed and Google scholar for articles published from January 1971 to December 2018, by using the terms “dietary Calcium Intake India”, “dietary calcium status India”, “calcium and Vitamin D India”. Studies which contained dietary calcium intake were taken for major consideration. For data on dietary calcium intake from 1970 till date, time trends were sourced from NIN-ICMR (national Institutes of Nutrition and Indian council of Medical Research) and NFI (nutrition foundation of India) websites. Most of the data was collected in previous searches of article on Modern India and Twin nutrient deficiency. Data was also sourced from National Sample Survey Office (NSSO)—Ministry of statistics and program implementation (MOSPI)-India, National Family Health Survey (NFHS) by International Institute of population sciences (IIPS), National dairy development board (NDDB) for data on milk consumption of India and FAO web site—Food and agricultural Organization of the United nations. Data was sourced from NINTATA website, Ministry of Human resources website. Searches were also conducted using the terms “Fortification of Milk India”, “RDA India, IOM, USA, and Canada”, “Nutrition apps”, “technology nutrition”, “mobile apps vitamin D calcium”, “mobile internet India”. Article resulting from these searches and relevant references cited in these articles were reviewed.

## Author Contributions

CH conceptualized study design and did relevant literature search, data collection, analysis, interpretation, preparation of figures, writing of the manuscript. HA was practically and intellectually involved in the strategy section including digital technologies for tackling calcium deficiency and role of corporate sectors, searched and reviewed relevant literature, participated in data collection, analysis and interpretation. ES was involved in development of figures, literature search for the article. All authors contributed to the article and approved the submitted version.

## Conflict of Interest

The authors declare that the research was conducted in the absence of any commercial or financial relationships that could be construed as a potential conflict of interest.
